# Synthetic Cannabis Overdose and Withdrawal in a Young Adult: A Case Report, Commentary on Regulation, and Review of the Literature

**DOI:** 10.1155/2016/3640549

**Published:** 2016-09-29

**Authors:** John Samaan, Gerardo F. Ferrer, Boye Akinyemi, Patricia Junquera, Juan Oms, Rhaisa Dumenigo

**Affiliations:** ^1^Department of Psychiatry, Larkin Community Hospital, South Miami, FL, USA; ^2^Department of Psychiatry and Behavioral Health, Jackson Memorial Health System, Miami, FL, USA

## Abstract

*Introduction*. Marijuana has been used for its psychotropic effects including enhanced relaxation and perceptual alterations. However, the use of synthetic marijuana (SM) leads to more frequent and drastic side effects than the typical use of regular marijuana, owing to the fact that SM has a shorter duration and an earlier peak of action. Despite all the potential adverse health effects associated with SM use, current health policies on SM are very limited. It is believed that the popularity of SM has increased, due to its easy accessibility in the US and lack of detection in typical urine drug screens for THC.* Case Report*. One case presented is of a young adult patient, with histories of recurrent synthetic cannabis and recreational cannabis use, who had developed drastic physiological and psychiatric symptoms, including the development of acute-onset psychosis.* Conclusion/Discussion*. This case, as many others nationwide, exemplifies the impact of synthetic cannabinoid use and abuse in adolescents. Side effects and adverse health consequences of synthetic cannabinoid use warrant stricter regulations and policies in order to decrease psychiatric hospital admissions and associated healthcare costs.

## 1. Introduction

Marijuana is used for its psychotropic effects including enhanced relaxation and perceptual alterations. The primary psychoactive ingredient found in marijuana is Δ-9 tetrahydrocannabinol (THC), which binds to endogenous cannabinoid receptors (CB1, CB2) [1]. Specifically, cannabis products including synthetic marijuana (SM) exert all their known psychotropic effects through the CB1 cannabinoid receptors. Such important classes of neurons that express high levels of CB1 receptors are GABAergic neurons in the hippocampus, amygdala, and the cerebral cortex [1]. Additionally, these neurons contain the neuropeptides cholecystokinin. In turn, when these cannabis products activate the CB1 receptors, the inhibition of the release of amino acids and monoamine neurotransmitters occurs. Further speaking, lipid derivatives, such as anandamide and 2-arachidonylglycerol, act as endogenous ligands for CB1 receptors (endocannabinoids). They may act as retrograde synaptic mediators of the phenomena of depolarization with possible induced suppression of inhibition or excitation in the hippocampus and cerebellum [2]. However, some SM products, such as JWH-015 and JWH-133, show affinity not only for the CB1 receptors, but also for the CB2 receptors. In turn, the SM products of JWH-015 and JWH-133, which show high affinity to CB2 receptors, may affect the immune system by modulating chemotaxis of T lymphocytes or inducing thymic atrophy and apoptosis [3]. More specifically, according to previous experimental research done, chronic exposure of mice to JWH-015 has been associated with increased vulnerability to drug abuse and depression [3]. Moreover, further research has shown that the SM product of JWH-133 was found to “dose-dependently” decrease the rewarding and locomotor stimulating effects of cocaine in mice [3]. These mentioned examples help to distinguish one of the many ways on how usage of SM products, such as JWH-015 and JWH-133, can differ in its effects when compared to the usage of traditional cannabis.

Additionally, these CB-2 receptors appear to be much present on the marginal zone of the spleen, tonsils, and immune cells, especially on macrophages, B cells, natural killer cells, monocytes, T lymphocytes, polymorphonuclear neutrophils, and astrocytes [4].

Of course, the locations of these CB receptors, as mentioned above, help determine what kind of underlying effects can occur, when these CB receptors are activated. For instance, as stated before, CB2 receptors are located throughout the immune system and related organs, such as the spleen, thymus gland, and tonsils [5]. It is also evident that CB2 receptors are found in greater concentrations throughout the gastrointestinal system, in which they appear to modulate intestinal inflammatory response. That being said, this is why patients that suffer from Crohn's Disease and Irritable Bowel Syndrome gain much relief from cannabis medicine [5]. Additionally, due to the CB2 receptors, it appears that cannabis has been shown to be useful for these conditions stated above, among other autoimmune diseases, in that nearly half of the cases of these diseases are put into full remission with the usage of cannabis [5].

On the other hand, when speaking on CB1 receptors, these receptors were shown to possess a very high binding affinity to the cannabinoid THC [5]. Moreover, these CB1 receptors appear to exist throughout the brain, central nervous system, connective tissues, gonads, glands, and related organs [5]. Due to the location of these receptors and their strong affinity to THC, consumption of cannabis strains and plants contacting high amounts of THC results in a relatively potent effect [5]. In turn, users of cannabis gain significant relief from pain, nausea, or depression while delivering a strong sense of euphoria [5].

Over the past few years, it has been apparent that there are hundreds of synthetic cannabis blends (often known as* synthetic marijuana* or, K2, or* Spice*) that have come to the market. Due to more and more banning of SM products and blends occurring in the society today, chemists have produced novel cannabimimetic designer drugs to replace SM products/blends. Currently, there are tens to hundreds of psychoactive cannabimimetic products in the market, the most infamous of which are JWH-018, JWH-073, JWH-200, CP-47,497, and cannabicyclohexanol [6] (please refer to Table 1 regarding further specific details and information on various types of SM products/blends).

Further speaking, significant resultant effects, from usage of these SM products, can be correlated when referring to the endogenous CB receptors and subsequent inhibition of specific amino acids, as stated above. For instance, SM products, as well as cannabis products, seem to have a direct vasodilator effect on cerebral blood vessels. It appears that smoking these products produces a dose-related increase in global cerebral blood flow in humans, which is consistent in the development of cerebral vasodilation. Cerebral vasodilation, in turn, may elicit profound hypotension [7]. Also, it should be noted that activation of CB1 receptors can have central side effects, such as ataxia and catalepsy. Additionally, due to the activation of these CB receptors, in particular CB2 receptors, selective CB2 receptor agonists have the potential to treat pain without eliciting the centrally mediated side effects [8].

More specifically, in regard to CB receptors and the inhibition of the release of amino acids and monoamine neurotransmitters, which follows the activation of these CB receptors, it appears that it has been shown to be effective for pain, as well for pain modulation.

To begin with, within the brain, CB1 receptors exert their effects mainly in the basal ganglia and the limbic system. Additionally, these receptors exert their effects in the cerebellum, as well as the male and female reproductive systems. When CB1 receptors are activated, they tend to selectively inhibit adenylate cyclase activity, which, consecutively, can affect perception, memory, and movement. Still, primarily, CB1 receptors are associated with euphoric and anticonvulsive effects, when bounded with the THC molecules, present in cannabis and synthetic marijuana. Other negative effects that are observed in certain susceptible individuals associated with CB1 receptor binding can include dysphoria and psychotomimetic effects as well [9].

Moving on to CB2 receptors, they very much resemble CB1 receptors in their structure and amino acid sequence. However, these two types of receptors are very different in terms of their activities and presence throughout the human body. For instance, CB2 receptors do maintain a crucial role in the immune system, inflammation, and, in particular, pain modulation [9]. Various studies that support the reduction in pain include case studies involving tactile and thermal allodyina, mechanical and thermal hyperalgesia, and writhing [9]. Furthermore, the location of the CB2 receptors in the microglia is particularly interesting within the scope of neuropathic pain research. These receptors located in the microglia support the theory around the benefits of cannabinoids in reducing cytokine-mediated neuroinflammation and in modulating neuropathic pain [9].

According to Rosenbaum et al., 2012, many of the SM-induced psychiatric effects consist of psychotic behavior and anxiety. Additionally, there is evidence that suggests that SM may trigger physical/psychological adverse effects similar to those of cannabis [10]. The use of SM leads to more frequent and drastic side effects than the typical use of regular marijuana, owing to the fact that SM has a shorter duration and an earlier peak of action [11]. SM use can lead to various adverse side effects including delusions, paranoia, hallucinations, anxiety, panic attacks, agitation, seizures, dizziness, and short-term cognitive deficits [12].

Moreover, SM can have significant effects in the neurons of the central nervous system (CNS). First off, it appears that the expression of CB receptors, in particular CB2 receptor messenger RNAs and proteins, is localized in the neurons of the brainstem. Subsequently, the CB2 receptors in brainstem appear to be activated by a CB2 receptor agonist, arachidonoylglycerol, and elevated by endogenous levels of endocannabinoids, which also act on CB1 receptors [13]. This, in turn, creates significant CNS effects, such as confusion, movement disorders, and agitation [13].

In addition to the expected CNS effects mentioned above, such as confusion, psychosis, agitation, loss of consciousness, and seizures, some of these SM based compounds have been associated with tachycardia, kidney damage, rhabdomyolysis, and even death.

Despite all the potential adverse health effects associated with SM use, current health policies on SM are very limited [14]. It is believed that the popularity of SM has increased, due to its easy accessibility in the US and lack of detection in typical urine drug screens for THC. Therefore, it is imperative to evaluate the impact of SM on mental health and more importantly the social and legal implications surrounding its use.

In lieu with SM and the health policies involved, there is evidence that action has been already taken to control the use of SM. To being with, as for epidemiologic studies involving the SM use, according to statistics from the National Institute of Drug Abuse (NIDA), in 2012, 11% of American High School Seniors used SM in that particular year; 14% of these seniors were male, while 8% were females [15]. Additionally, as stated in the NIDA, there were a total of 11,406 Emergency Room (ER) visits in 2010 due to the use of SM [15]. Of these 11,406 ER visits, 75% of these individuals were adolescents and young adults from the ages of 12 to 29 [15]. Also, 22.5% of the ER visits involved females and 77.5% involved males [15].

Additionally, according to “A Survey of Synthetic Cannabinoid Consumption by Current Cannabis Users” (Gunderson et al., 2014), collaborators found that, with those individuals who are exposed to cannabis and tobacco, nearly all (91%) were familiar with SM products, half (50%) reported smoking SM products previously, and a considerable minority (24%) reported current use of SM (within the prior month) [16].

As for legislation, such as legal consequences for manufacturing and disturbing SM products, since 2011, all 50 states have banned synthetic drugs, including synthetic cannabinoids (a.k.a. synthetic marijuana, K2, spice) and cathinones (a.k.a. bath salts) [16]. In the beginning, state legislative action targeted specific versions of these drugs with individual bans. However, since chemists are now slightly changing the chemical composition of these substances to create new but very similar drugs, not covered by law, legistlation, in recent years, is targeting entire classes of substanes, in which broad language is used to label the prohibited drugs. In turn, the intent of the general ban is to prevent new forms of synthetic cannabinoids and other synthetic drugs from remaining unregulated [17].

For example, a few states have passed laws restricting marketing, displaying, labeling, and advertising of these substances by exploiting consumer protection laws or classifying these activities as “deceptive trade practices.” Additionally, law makers and enforcers are using existing provisions such as agricultural regulations, consumer protection laws, and public nuisance laws to penalize those selling these drugs [17].

Consequently, this case illustrates how easy accessibility and limited regulation on SM adversely impact mental health thus posing a challenging problem for psychiatrists nationwide. The present case report leads to consideration of the critical need for regulations and effective toxicology screens for SM.

## 2. Case Report

An 18-year-old Hispanic male was brought to our emergency department by his parents after five days of acute-onset auditory hallucinations, paranoid delusions, and, per the mother, symptoms of panic attacks, including palpations, shortness of breath, diaphoresis, chest tightness, and hand numbness. The patient presented with impulsivity and agitation and stated he had suicidal thoughts. Additionally, the patient reported a history of cannabis abuse and, more recently, synthetic marijuana (SM)/cannabis use (3-4 days prior to presentation), which were purchased from internet blog sites and convenience stores.

It was apparent that the onset of psychosis coincided with the patient's most recent SM use. Of note, the patient did not have any previous psychiatric history, nor a psychotic episode, prior to his synthetic marijuana use. Nevertheless, an antipsychotic regimen was provided to the patient during his hospitalization which was beneficial in controlling his transient acute psychosis. Moreover, a urine drug screen in the ED was negative for THC, which fortified the patient's history of not using regular marijuana since a month prior to hospitalization.

Thereafter, a three-month follow-up phone call made to the patient revealed that his mental health had been progressively improving after discharge and, at that moment, he was doing very well. The patient mentioned that the symptoms subsided several weeks (2-3 weeks) after his discharge from the hospital. During that time, the patient refrained from any illicit drug use, including SM use. The patient remained under the care of a licensed psychiatrist throughout the period after inpatient treatment. According to the patient, compliance was maintained regarding attendance of sessions with his outpatient psychiatrist.

## 3. Discussion

It is of utmost importance to note that there may be some differences between a psychotic episode secondary to synthetic cannabis/marijuana use and an exacerbation of a psychotic episode due to a defined psychiatric illness. For instance, synthetic cannabis/marijuana products can induce a brief acute psychotic state that can subside within several days to weeks, after cessation of the drug, in individuals with no previous diagnosis of a psychiatric disorder. Still, synthetic cannabis/marijuana products can trigger psychosis in individuals who have a current diagnosis of psychosis. In fact, further use of these products can worsen psychotic symptoms in those individuals with a current diagnosis of psychosis or specific type of a psychotic disorder. It is therefore very ideal to obtain a thorough psychiatric history, a complete mental status assessment, and an appropriate follow-up regarding a patient before determining the etiology of an acute psychotic episode. This would help define if an acute psychotic episode may be secondary to a substance (in this case SM) or secondary to a chronic psychiatric illness (e.g., Schizophrenia Spectrum Disorders, Bipolar Disorder, and Major Depressive Disorder with Psychotic Features). That being said, certain lab findings alone, more specifically a negative urine drug screen for THC, will not make the determination on whether the psychosis is secondary to SM or if it is secondary to a chronic mental illness [1].

To better clarify how to differentiate between a “first break” psychotic episode and a substance-induced psychotic episode, several demographic, familial, and clinical characteristics haven to be taken into consideration. According to study done by Caton et al., “Differences Between Early-Phase Primary Psychotic Disorders with Concurrent Substance Use and Substance-Induced Psychoses,” there are various aspects that need to be looked upon, when comparing those with a primary psychotic disorder/episode and those with a substance-induced psychotic disorder. For instance, it was evident, from the above the study, that patients with a substance-induced psychotic disorder had a significantly later age of onset of psychosis, greater conjugal ties, greater antisocial personality disorder comorbidity, more frequent homelessness, and poorer family support, and more patients had at least one parent with a substance abuse problem [25]. Additionally, collaborators from the study concluded that patients with primary psychosis, or a primary psychotic episode, had more severe psychiatric symptoms associated with less insight, a finding that is not limited to positive symptoms but also includes negative symptoms [25]. Still, on the other hand, subjects with substance-induced psychosis had more severe forms of substance use disorders, characterized by long periods of substance use, severe psychosocial problems, and greater dependence [25]. Furthermore, another characteristic distinguishing a substance-induced psychotic break, from an acute psychotic episode/break, was the existence of a significant amount of visual hallucinations [25].

Although these two types of psychotic episodes, mentioned above, may differ in many aspects, only a few features can account for their differences. For instance, it is evident that parental substance abuse, a concurrent diagnosis of drug dependence, and the appearance of visual hallucinations are greater in patients with a substance-induced psychotic disorder [25]. On the other hand, higher levels of psychiatric symptoms seem to be more evident in a primary “first break” psychotic disorder [25].

In regard to these “higher level of psychiatric symptoms,” these can include a variety of Kurt Schneider's “first-rank symptoms,” such as audible thoughts (hearing voices discussing about oneself), normal perception followed by delusionally personalized interpretation, thought insertion, withdrawal and broadcasting, and somatic passivity (in other words, experiencing one's emotions or impulses as being controlled by an external force) [26].

As for SM-induced psychotic episodes, there have been various case studies, just as the one discussed above, that share similar and almost identical presentations. For instance, there have been reports that SM products can have urgent adverse effects, including tachycardia, agitation, excess sedation, and loss of consciousness. In addition, reports of psychosis associated with SM products have emerged since 2010. Among them, a number of psychotic symptoms are described in patients ranging in age from adolescence to adulthood, both with and without histories of psychosis [27].

Overall, there are various reports suggesting that synthetic cannabinoid (or SM) intoxication is associated with acute psychosis as well as exacerbations of previously stable psychotic disorders. Also, SM intoxication may have an inclination to trigger a chronic psychotic disorder among individuals that appear vulnerable [27]. For instance, for vulnerable individuals, such as patients that have been diagnosed with a Schizophrenia Spectrum Disorder, a history of SM and cannabis use, with these patients, appears to be associated with a chronic psychotic episode onset 2 to 3 years earlier compared with nonusers [27]. In addition, SM and cannabis can be a definite risk factor for conversion to psychosis in some studies of prodromal schizophrenia [27]. Specifically, when we speak in terms of prodromal schizophrenia, we are referring to the common signs and symptoms in the early stages of schizophrenia, such as reduced concentration and attention, reduced drive and motivation, depressed mood, sleep disturbances, anxiety, social withdrawal, suspiciousness, irritability, and deterioration in role functioning [26].

Speaking in regard to a transient acute psychotic episode caused by SM use, the cessation of use will bring on resolution of the psychosis. The short-term use of an antipsychotic or a benzodiazepine regimen may be warranted depending on the level of distress. Additionally, it has been shown that psychoeducation and Cognitive Behavioral Therapy (CBT) have been successful in reducing SM use in various patients experiencing their first episode of psychosis. Moreover, further research has specifically shown Clozapine rather than Risperidone as being more effective in managing acute psychotic symptoms secondary to SM use [1].

Users of SM products appear to have a variety of physical effects ranging from nausea to more serious sympathomimetic-like symptoms, such as psychomotor agitations, abnormal vital signs, including hypertension and tachycardia, diaphoresis, and palpitations, as mentioned previously [26]. Also, even though infrequently associated with side effects from smoking SM, clinical case reports have described generalized convulsions secondary to the usage of 4 different synthetic cannabis derivatives, JWH-018, JWH-081, JWH-250, and AM-2201 [2].

Moreover, there have been various case reports of acute psychosis that has been caused by the usage of specific derivatives of synthetic cannabis. For instance, the most common derivative of synthetic cannabis, JWH-018 (a.k.a. spice), has been very much associated with anxiety, exacerbation of paranoid delusions, delusions of control, auditory and visual hallucinations, agitation, disorganization, Capgras delusions, confusion, and ideas of reference, as well as tachycardia and hypokalemia [27]. Additionally, a second derivate of synthetic cannabis, CP-47,497, was also shown to cause agitation, disorganization, paranoia, and grandiose delusions [27]. Also, with the JWH-018 and CP-47,497 derivatives, it has been known that these products can even trigger an exacerbation of psychosis for those who have currently been diagnosed with a psychotic disorder [27].

## 4. Further Conclusions

Synthetic marijuana use is insidiously becoming a mental illness concern given its appreciable contributions to acute onset and exacerbation of existing psychiatric conditions.

However, there has been a recent increment in the level of awareness for marijuana use for medical purposes in the United States and internationally, which tends to juxtapose regulation of cannabinoids and SM. For instance, there have been reports that cannabinoids are to be of therapeutic value in neurological disorders, associated with spasticity, ataxia, and muscle weakness. Also, it appears that the efficacy of THC is equivalent to codeine, making cannabinoids have an adjunctive and a promising role in the management of pain [28]. Additionally, other therapeutic uses of cannabinoids include the following: antiemetic use, appetite stimulation, treatment of epilepsy, treatment of glaucoma, treatment of bronchial asthma, and they can serve as treatment for alcohol and opiate withdrawal [28].

Moreover, therapeutic advantages can occur with the usage of SM and synthetic cannabinoids as well. According to a study done by Darmani, he had used animal models (shrews) to discover that synthetic cannabinoid analogs, specifically nabilone and levonantradol, can prevent emesis in cancer patients receiving chemotherapy [29]. Yet another study from Brents et al. had revealed that synthetic cannabinoid derivatives, in particular JWH-018 and JWH-073, had contained analgesic properties. Additionally, as evident from the results of this study, just mentioned in the previous sentence, researchers found that JWH-018 and JWH-073 derivatives had shed insight into potential drug abuse liability of SM use, overall [30].

Despite the therapeutic advantages of cannabis and SM, there is still a need for strict regulations and control measures in defining limits for recreational use, due to its severe potential psychiatric adverse effects. Overall, it appears that the risks and the adverse effects from SM usage appear to extremely outweigh the therapeutic advantages of SM, based on what has been mentioned already regarding the use and addiction of SM. That being said, legalizations and further laws should ban the overall sales of SM, along with the production of SM. In fact, implementation of measures and regulations of the usage of SM has been already introduced, in the United States, as mentioned earlier.

Looking back at the case study mentioned above, we find that the epidemiology, as well as the need for legislation, can contextualize with the patient's account. For instance, as stated in the Introduction, it was found that about 10% of high school seniors, the majority being males, had experimented with SM in 2012 [31]. Additionally, once again, as described in the Introduction, approximately 11, 406 ER visits, in 2010 were cases due to intoxication and/or withdrawal of SM. Of these 11, 406 visits, 75% were adolescents, aged 12–29 [31]. As one can see, these mentioned findings appear to very much relate to the case study description: the patient was just finishing high school and he was a Hispanic male at the age of 18, at the time.

Furthermore, if we look at the legislation for the production, the possession, and the usage of SM, it appears that much needs to be done to promote stricter regulation. As with our case study, the patient stated that he had obtained these SM products from “internet sites and convenience stores.” Keeping that in mind, it seemed that SM products were easily accessible, at the time. Thus, a need for further severe restrictions, on the sale of SM, could possibly be one way to reduce psychiatric hospital admissions due to SM intoxication/withdrawal.

Moreover, SM, specifically, appears to be more injurious to users' health given its potency and affinity for cannabinoid receptors. For instance, if we look back at our case study, we see that the patient did not just exhibit transient acute psychosis, but, as well, the patient had been experiencing panic attacks, palpations, shortness of breath, diaphoresis, chest tightness, hand numbness, agitation, and impulsivity, at the time. That being said, it appears that he biochemical nature of SM, along with its strong affinity/potency to CB1 and CB 2 receptors (as described in the Introduction), appears to be the cause responsible for the development of these psychotic and physical adverse effects, as we see in this case study.

Therefore, there is a need for regulations that would abolish the production and sale of synthetic marijuana given its hazardous implications. Also, there is a need for these specific regulations due to the ample availability of constantly changing synthetic marijuana formulations, in an attempt to break set substance laws. In review thus far, there is no benefit of synthetic marijuana over traditional cannabis. However, SM has more injurious medical and psychiatric implications when compared to traditional cannabis as documented in this case report.

Nonetheless, there is a further need for production regulations, including standardization and monitoring designed structural compounds of SM, in order to create the pathway to the formulation of effective toxicology screening techniques and, in turn, limiting the use of SM, overall.

## Figures and Tables

**Table 1:** Cannabis characteristics versus synthetic marijuana (SM) characteristics: similarities and differences.

Topic of concern	Cannabis characteristics	Synthetic marijuana (SM) characteristics
Adverse side effects	According to Karant, the following effects occur with cannabis use [18]: (i) Elevated mood (ii) Relaxation (iii) Altered perception of surrounding conditions (iv) Symptoms of psychosis (delusions, disorganized thinking, and detachment from reality) (v) Cognitive deficits during acute intoxication (vi) Precipitation of onset or relapse of schizophrenia (vii) Cannabis dependence	According to Freeman et al., 2013 [19], the following side effects could occur with SM use: (i) Ischemic stroke (ii) Elevated mood (iii) Relaxation (iv) Altered perception of surrounding conditions (v) Extreme psychosis (delusions, disorganized thinking, and detachment from reality to a great extent) (vi) Violent behavior and suicidal thoughts (vii) Tachycardia (viii) Severe abdominal pain, nausea, and vomiting that occurs over several months and resolves when SM use has been discontinued (known as synthetic cannabinoid hyperemesis syndrome)

Mechanism of action	(i) It binds to cannabinoid receptors in the brains and other organs as the endogenous ligand, anandamide [20]	(i) It binds to cannabinoid-like receptors in the brains and other organs as the endogenous ligand, anandamide [20] (ii) Synthetic cannabinoids and other identical products seem to have a similar mode of action as marijuana, in which these substances have affinity to various cannabinoid receptors (e.g., CB1 and CB2) [20] Synthetic cannabinoids (“spice”) fall into seven major structural groups: Note that spice has high potency and high affinity for cannabinoid receptors thus making it very efficacious [21]: (i) Naphthoylindoles (e.g., JWH-018, JWH-073, and JWH-398) (ii) Naphthylmethylindoles (iii) Naphthoylpyrroles (iv) Naphthylmethylindenes (v) Phenylacetylindoles (i.e., benzoylindoles, e.g., JWH-250) (vi) Cyclohexylphenols (e.g., CP-47,497 and homologues of CP-47,497) (vii) Classical cannabinoids (e.g., HU-210)

Availability	(i) Remains illegal in most parts of the United States; State laws determine the legality of marijuana [22] (ii) Available illegally in most US states	(i) It is readily available in the United States because manufacturers are able to bypass the law by changing chemical compounds that have been outlawed thus creating various cannabinoid-like substances which have not been studied for side effects [21] (ii) In most countries around the world, including the United States, synthetic cannabis is illegal (iii) Cannabinoids are becoming more readily available in the United States due to articles and documentaries showing its benefits in regulating mood and also control of epileptiform activities [23]

Screening & Detection	(i) THC compound can be detected consistently by simple serum, urine, or oral swab tests [24] (ii) Compound can be detected for up to 5–7 days depending on body fluid obtained and quantity of cannabis exposure [24]	(i) Newly developed laboratory tests are able to successfully detect up to 20 synthetic cannabinoid substances; due to the constant changes made to the compounds, it is difficult to have a single test determining the presence of synthetic cannabinoids in body fluids [24] (ii) New advances have shown that liquid chromatography/tandem mass spectrometry (LC-MS/MS) and gas chromatography/mass spectrometry (GC/MS) can detect several metabolites of synthetic cannabinoid substances in the urine: JWH-018 and JWH-073, along with other various metabolites [24] (iii) Compounds can be detected for up to 2-3 days based on immunoassay utilized and quantity of synthetic cannabinoid exposure [24]
